# Outcomes associated with planned place of birth among low-risk pregnancies in Ontario, Canada (2012–2021): A protocol for a population-based propensity score weighted cohort study

**DOI:** 10.1371/journal.pone.0302489

**Published:** 2024-05-13

**Authors:** Elizabeth K. Darling, Vanessa Hébert, Giulia Muraca, Angela Reitsma

**Affiliations:** 1 McMaster Midwifery Research Centre, McMaster University, Hamilton, Ontario, Canada; 2 Department of Obstetrics and Gynecology, McMaster University, Hamilton, Ontario, Canada; 3 Department of Health Research Methods, Evidence, and Impact, McMaster University, Hamilton, Ontario, Canada; PLOS: Public Library of Science, UNITED KINGDOM

## Abstract

**Background:**

Evidence suggests that for low-risk pregnancies, planned home births attended by a skilled health professional in settings where such services are well integrated are associated with lower risk of intrapartum interventions and no increase in adverse health outcomes. Monitoring and updating evidence on the safety of planned home births is necessary to inform ongoing clinical and policy decisions.

**Methods:**

This protocol describes a population-based retrospective cohort study which aims to compare risk of (a) neonatal morbidity and mortality, and (b) maternal outcomes and birth interventions, between people at low obstetrical risk with a planned home birth with a midwife, a planned a hospital birth with a midwife, or a planned hospital birth with a physician. The study population will include Ontario residents who gave birth in Ontario, Canada between April 1, 2012, and March 31, 2021. We will use data collected prospectively in a provincial perinatal data registry. The primary outcome will be severe neonatal morbidity or mortality, a composite binary outcome that includes one or more of the following conditions: stillbirth during the intrapartum period, neonatal death (death of a liveborn infant in the first 28 completed days of life), five-minute Apgar score <4, or infant resuscitation requiring cardiac compressions. We will conduct a stratified analysis with three strata: nulliparous, parous—no previous caesarean birth, and parous—prior caesarean birth. To reduce the impact of selection bias in estimating the effect of planned place of birth on neonatal and maternal outcomes, we will use propensity score (PS) overlap weighting (OW) and modified Poisson regression to conduct multivariate analyses.

## Introduction

Midwifery has been a regulated, publicly funded health profession in Ontario since 1994 [1]. In 2020–21, midwives attended 20% of all the births in Ontario, and approximately 16% of these births were out-of-hospital, either at home (14%) or in a free-standing birth centre (2%) [2]. While hospital birth is frequently perceived as safer than home birth, evidence suggests that for low risk pregnancies under the care of trained providers working in a health care system where home birth services are well integrated, planned hospital birth may increase the use of intrapartum interventions, including caesarean delivery, without improving health outcomes [3–5]. Two cohort studies published by Hutton et al. reported no differences in perinatal/neonatal outcomes between planned home and planned hospital birth under midwifery care in Ontario between 2003–2009 [3, 4]. A systematic review of evidence from studies reporting on over 500,000 births showed no significant increase in the risk of adverse perinatal or neonatal mortality with planned home births in health systems where home birth is well integrated [6].

Health professionals, pregnant people, and policy makers rely on research evidence to inform decisions regarding home birth [7]. Questions about the safety of home birth are a critical barrier to those who are undecided about where to give birth [7].Variations in homebirth outcomes across different settings highlight that contextual factors may influence outcomes [8]. Given changes in contextual factors that may occur over time within a single jurisdiction, including changes in baseline health of birthing people, the volume of homebirth experience that midwives have, and the integration of home birth services within the health system, it is important to continuously monitor and update evidence on the safety of planned home births.

The availability of routinely collected perinatal data on all births in Ontario from 2012 onward though the Better Outcomes Registry and Network (BORN) provides a new opportunity for home birth research [8]. While the previous research on planned home births in Ontario used a midwife-attended planned hospital birth comparison group, the BORN perinatal registry data now allows for inclusion of a physician-led care comparison group. We also plan to address the methodological limitations of previous research (i.e., potential confounding and selection bias) by using statistical methods not previously used in home birth studies.

### Objectives

This study aims to compare risk of (a) neonatal morbidity and mortality, and (b) maternal outcomes and birth interventions, between people at low obstetrical risk with a planned home birth with a midwife, a planned a hospital birth with a midwife, or a planned hospital birth with a physician.

We hypothesize that that we will find no statistically significant difference between groups in the frequency of neonatal morbidity and mortality and severe maternal morbidity, and that there will be statistically significant lower rates of obstetrical interventions among the group that plans to give birth at home. Findings from this study will help midwives inform clients about the safety of planned home births, allow clients to make informed decisions about their choice of birthplace, and support evidence-informed policy and regulation pertaining to the choice of home birth in Ontario.

## Methods

### Study design and population

We will conduct a population-based retrospective cohort study of all Ontario residents who gave birth in Ontario between April 1, 2012, and March 31, 2021, using linked administrative data sets. The data sets to be used are summarized in Table 1 and described in further detail in the ’Data sources’ section below.

**Table 1:** Data sources.

Data Source	Full Name	Description
BORN BIS	Better Outcomes Registry and Network Information System	A provincial perinatal registry that was established in 2012 to collect data about every birth in Ontario, as well as data about pregnancy and the early childhood period.
RPDB	Registered Persons Database	An ICES derived dataset containing demographic information about anyone who has ever had an Ontario health insurance plan number. Based on data from the Ministry of Health and enhanced with other ICES data holdings, including CIHI-DAD and NACRS.
PCCF+	Postal Code Conversion File Plus	A macro that converts Canadian postal codes to Statistics Canada geographical areas and allows linkage to area-based census data.
ON-MARG	Ontario Marginalization Index	An area-based index, derived using factor analysis of census data, that measures four dimensions of marginalization: residential instability, material deprivation, dependency, and ethnic concentration.
INST	Institution Information System	A set of linkable datasets containing information about Ontario health care institutions funded by the Ministry of Health.

We will exclude individuals with pregnancies ending in miscarriages <20 weeks’ gestation, induced abortions, or fetal death occurring before labour, as well as records of individuals who were discharged from midwifery care prior to giving birth. We will also exclude records that are missing values for planned stratification (i.e., parity, number of previous caesarean deliveries), exposure classification (planned place of birth for midwifery billable courses of care), or primary outcome assessment (stillbirth, neonatal death, or timing of fetal death).

**Fig 1:**
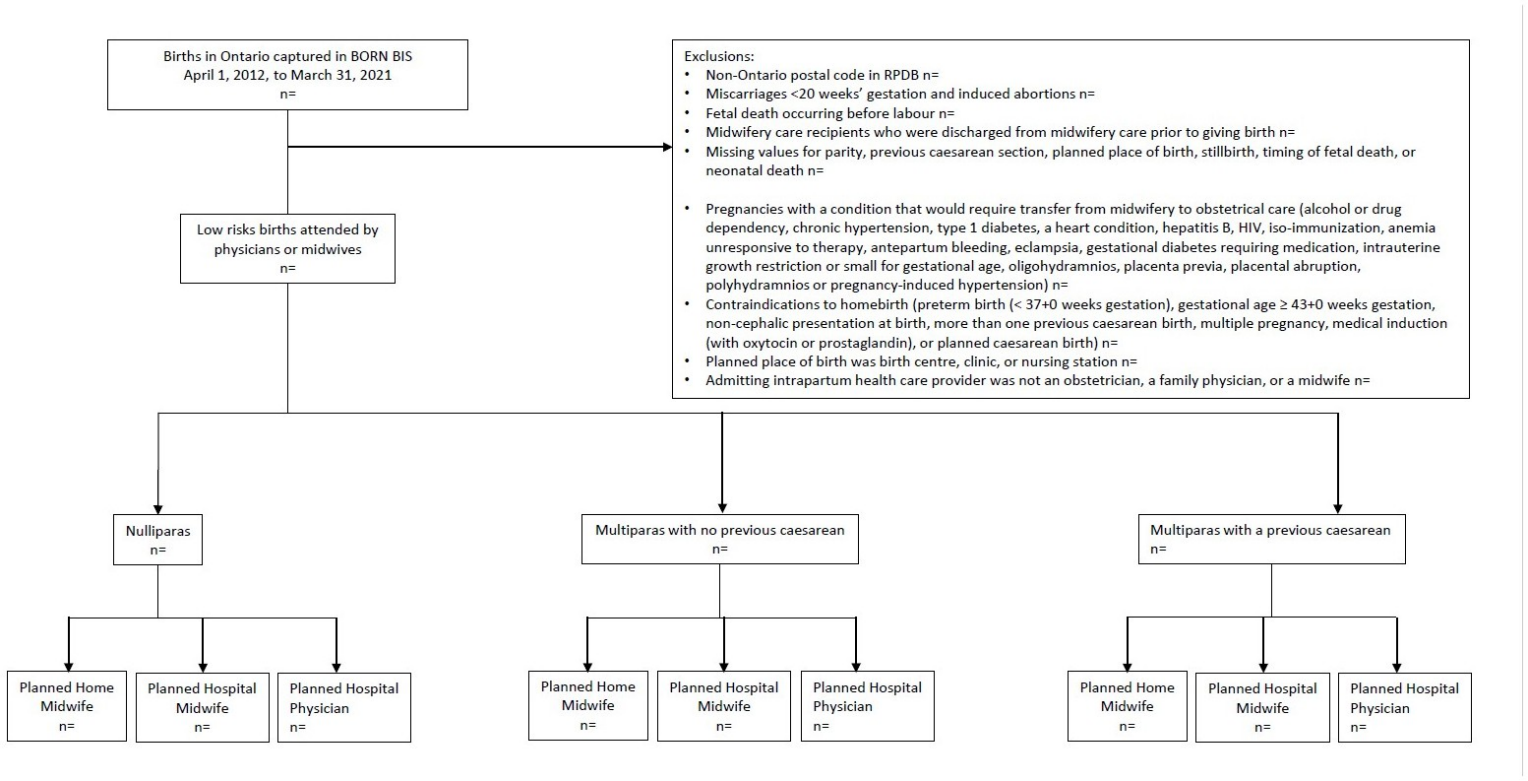
Study flow diagram.

To ensure that the entire study population is restricted to people who would be considered candidates for home birth, we will exclude records that reported a condition or complication that would historically have been classified as a mandatory antenatal consultation or transfer of care to a physician according to the College of Midwives of Ontario (i.e., records that indicated alcohol or drug dependency, chronic hypertension, type 1 diabetes, a heart condition, hepatitis B, HIV, iso-immunization, anemia unresponsive to therapy, antepartum bleeding, eclampsia, gestational diabetes requiring medication, intrauterine growth restriction or small for gestational age, oligohydramnios, placenta previa, placental abruption, polyhydramnios or pregnancy-induced hypertension) [9]. We will also exclude individuals with the following contraindications to home birth: preterm birth (< 37 weeks gestation), gestational age ≥ 43 weeks gestation, non-cephalic presentation at birth, multiple pregnancy, more than one previous caesarean birth, medical induction (with oxytocin or prostaglandin), or planned caesarean birth [10]. We will exclude births involving newborns with major congenital anomalies, as listed by the Canadian Neonatal Network [11]. We will exclude births planned to occur in birth centres and clinics, and births where the admitting health care provider is not an obstetrician, a family physician, or a midwife. We will stratify the population into three groups: nulliparas, multiparas, and multiparas with a previous caesarean section. Fig 1 presents the study flow diagram. Details regarding the data sources and codes for the creation of the cohort are described in Table 2.

**Table 2:** Data used to create cohort, demographic characteristics, exposure, outcomes, and covariates.

Purpose	Concept	Data source	Variables and codes
**Inclusion criteria**	Birth in Ontario between April 1, 2012 and March 31, 2021	BIS	pregnancy_id
**Exclusion criteria**	Non-Ontario resident based on postal code or LHIN on date of the included birth	BIS and RPDB	b_bdate from BIS; If in RPDB: substr(prcddablk,1,2) ne ‘35’; If not in RPDB: maternal_residence_LHIN_code_num IN (-1) in BORN.AGG_PREGNANCY
	Miscarriage <20 weeks or induced abortion at any gestational age	BIS	pregnancy_outcome_AG_id IN (1021035, 1021040, 1021050, 1021070) in BORN.MW_PREGNANCY_COC or agg_pregnancy_outcome_ID in (1021035, 1021040, 1021050, 1021070) in BORN.AGG_PREGNANCY, or neonatal_death_id = 1018210 in BORN.AGG_INFANT
	Fetal death before labour	BIS	pregnancy_outcome_AG_id = 1021080 in BORN.MW_PREGNANCY_COC or agg_pregnancy_outcome_ID = 1021080 in BORN.AGG_Pregnancy datasets
	Midwifery care recipient not under midwifery care at the time of birth	BIS	Midwifery care recipients: only apply the exclusions below to records with pregnancy_enc_id in BORN.MW_ PREGNANCY_COC Exclude if either of the following variables = yes: MW—Discharge from care during pregnancy = yes Variable MWdischfrmcareduringpregflg = ‘Y’ in BORN.MW_PREGNANCY_COC or BORN.AGG_Pregnancy datasets MW—Unreturned transfer of care = yes Variable unret_transfer_of_care_AG = ‘Y’ in MW_PREGNANCY_COC or BORN.Antenatal_General [Note: Do not use variable unret_transfer_of_care_BM = ‘Y’ and do not use variable unret_transfer_of_care_PPM = ‘Y’]
	Missing data on parity	BIS	• Parity missing in BORN.AGG_Pregnancy AND missing in BORN.MW_PREGNANCY_COC
	Missing data on number of previous cesarean deliveries	BIS	If Parity = 0 then not missing Else (i.e., if Parity ≥ 1) if num_of_pre_cs_births missing in BORN.AGG_Pregnancy AND num_of_pre_cs_births missing in BORN.MW_PREGNANCY_COC
	Missing data on planned place of birth for midwifery care recipients only	BIS	• Midwifery care recipients: only apply the exclusions below to records with pregnancy_enc_id in BORN.MW_ PREGNANCY_COC • MW_plan_loc_of_birth_id NOT IN (1018010, 1018020, 1028022, 1018024, 1018030, 1018040, 3000165) in AGG_Pregnancy AND MW_plan_loc_of_birth_id NOT IN (1018010, 1018020, 1028022, 1018024, 1018030, 1018040, 3000165) in BORN.MW_PREGNANCY_COC
	Missing data on stillbirth or neonatal death	BIS	• pregnancy_outcome_AG_id missing in MW_PREGNANCY_COC AND agg_pregnancy_outcome_ID missing in AGG_Pregnancy datasets
	Missing data on timing of stillbirth	BIS	• agg_pregnancy_outcome_ID = 1021060 in AGG_Pregnancy
	Condition classified as requiring physician care according to the College of Midwives of Ontario	BIS	includes any of the following found in BORN.MW_pregnancy_COC or BORN.AG or BORN.AGG_pregnancy or BORN.AS or BORN.LBM: mat_pre_exist_health_cond_id (M0013F) IN (1016600, 1016610, 1016640, 1016650, 1016660, 1016670, 1016680, 1016740, 1016750, 1016970, 1016980, 1016990) in any dataset infection_id (D0017F) = 1020940 in any dataset diabetes_and_pregnancy_id (D0013F) IN (1013440, 1013450, 1013460, 1013465, 1013490, 1013500, 1013520, 1013530, 1013540, 1013545) in any dataset preg_hypertension_disorder_id (D0016F) IN (1020800, 1020820, 1020840, 1020850, 1020854) in any dataset complication_id (M0531F) = 1020290 in any dataset MW—antenatal_consult_reason_id (D0107F) IN (1063250, 1063260, 1063270, 1063280, 1063290, 1063300, 1063310) (only availably in BORN.AG and BORN.MW_PREGNANCY_COC datasets)
	Preterm birth (<37 weeks gestation) or missing gestational age	BIS	GA_at_birth_weeks <37 or missing in BORN.AGG_PREGNANCY dataset
	Gestational age ≥ 43 weeks gestation	BIS	GA_at_birth_weeks >42 in BORN.AGG_PREGNANCY dataset
	Non-cephalic presentation at onset of labour	BIS	• Presentation_type_id IN (1021100, 1021110, 1021120, 1021130, 1021140, 1021200, 3000089) from BORN.MW_pregnancy_COC or BORN.MW_BIRTH_COC or BORN.AGG_PREGNANCY datasets
	Multiple pregnancy	BIS	NUMBER_OF_FETUSES >1 from BORN.MW_Pregnancy_COC or BORN.AGG_PREGNANCY datasets Or Consult_Reason_id = 1025340 in BORN.MW_pregnancy_COC_consult dataset Or pregnancy_id links to more than one baby record in BORN.AGG_INFANT
	More than one previous caesarean section	BIS	• num_of_pre_cs_births >1 from BORN.AGG_Pregnancy or BORN.MW_PREGNANCY_COC
	Pharmacological induction of labour	BIS	• labour_induction_method_id IN (1014620, 1014625, 3000008) from BORN.AGG_Pregnancy or BORN.MW_PREGNANCY_COC
	Planned cesarean birth	BIS	Birth_Type_ID = 1012900 from BORN.MW_pregnancy_COC or BORN.AGG_PREGNANCY datasets Or CS_type_id IN (1013360, 1013370, 1048727) from BORN.MW_pregnancy_COC or BORN.AGG_pregnancy datasets
	Severe congenital anomaly (major anomalies as per CNN)	BIS	Any of the following from BORN.AGG_infant: Newborn_Anomalies_Confirmed_ID (D0025) IN (1009400, 1009540, 1009630, 1009600, 1009640, 1009930, 1009940, 1006890, 1007350, 1006670, 1007310, 1007000, 1007300, 1007320, 1006760, 1012160, 1012150, 1012130, 1009230, 1009330, 1009060, 1009310, 1009005, 1009110, 1009150, 1009070, 1008890, 1008900, 1008920, 1008680, 1008690, 1008860, 1008740, 1008790, 1008870, 1007960, 1007950, 1009550, 1012170, 1006520, 1006500, 1010510, 1011970, 1007650, 1007660, 1007670, 1007640, 1007630, 1007480, 1007490, 1007500, 1007610, 1007620, 1007680, 1007530, 1007540, 1007550, 1007470) NB_ANOMALY_ICD10_CONFIRMED_ID IN (3101375, 3101126, 3101128, 3101132, 3101376, 3101131, 3101135, 3101144, 3101145, 3101181, 3101198, 3101175, 3101196, 3101183, 3101177, 3101197, 3101165, 3101166, 3101164, 3101403, 3101209, 3101404, 3101212, 3101213, 3101214, 3101216, 3101406, 3101217, 3101217, 3101218, 3101238, 3101228, 3101411, 3101229, 3101269, 3101412, 3101222, 3101223, 3101224, 3101413, 3101221, 3101410, 3101234, 3101233, 3101253, 3101256, 3101257, 3101254, 3101249, 3101167, 3101206, 3101205, 3101430, 3101278, 3101280, 3101279, 3101289, 3101285, 3101288, 3101286, 3101281, 3101284, 3101282, 3101283)
	Planned place of birth was other, undecided, birth centre, clinic, or nursing station (keep if planned home or planned hospital)	BIS	Only apply this exclusion to midwifery care recipients, i.e., records with pregnancy_enc_id in BORN.MW_ PREGNANCY_COC: • KEEP if MW_PLAN_LOC_OF_BIRTH_ID in (1018010, 1018020) from BORN.MW_pregnancy_COC or BORN.AGG_pregnancy datasets
	Admitting health care provider was not an obstetrician, a family physician, or a midwife	BIS	• KEEP if Intrapartum_Admission_hcp_id in (1014400, 1014410, 1014420) from BORN.LBM or BORN.MW_pregnancy_COC datasets
**Main Exposure**	Planned place of birth	BIS	All records that do not have pregnancy_enc_id in BORN.MW_ PREGNANCY_COC will = hospital If record has pregnancy_enc_id in BORN.MW_ PREGNANCY_COC: = home if MW_PLAN_LOC_OF_BIRTH_ID in (1018010, 1018030, 1018040) from BORN.MW_pregnancy_COC or BORN.AGG_pregnancy = hospital if MW_PLAN_LOC_OF_BIRTH_ID in (1018020) from BORN.MW_pregnancy_COC or BORN.AGG_pregnancy
	Planned place of birth/provider groups	BIS	Three exposure groups: 1. Planned home with a midwife: • if MW_billability_type_ID in (1017760, 1017770) from BORN.AG or BORN.LBM or BORN.MW_PREGNANCY_COC or BORN.PPM AND MW_PLAN_LOC_OF_BIRTH_ID in (1018010) from MW_pregnancy_COC and AGG_pregnancy datasets 2. Planned hospital with a midwife: • if MW_billability_type_ID in (1017760, 1017770) from BORN.AG or BORN.LBM or BORN.MW_PREGNANCY_COC or BORN.PPM AND MW_PLAN_LOC_OF_BIRTH_ID in (1018020) from MW_pregnancy_COC and AGG_pregnancy datasets 3. Planned hospital with a physician: • All remaining records in the cohort
**Stratification**	Parity/Previous cesarean	BIS	Three strata based on combination of Parity and Previous caesarean: 1. Nulliparous: If parity = 0 (from BORN.AGG_PREGNANCY or BORN.MW_PREGNANCY_COC) 2. Multiparous—no previous cesarean: If parity > 0 and num_of_pre_cs_births = 0 (from BORN.AGG_ PREGNANCY or BORN.MW_PREGNANCY_COC) 3. Multiparous—previous cesarean: If parity > 0 and num_of_pre_cs_births = 1 (from BORN.AGG_ PREGNANCY or BORN.MW_PREGNANCY_COC)
**Primary Outcome**	Severe neonatal morbidity or mortality	BIS and RPDB	Composite binary outcome will be ‘yes’ if one or more of the following: stillbirth during intrapartum period—pregnancy_outcome_id = ‘1021090’ in BORN.AGG_INFANT neonatal death (death of a liveborn infant in the first 28 completed days of life)–neonatal_death_id = 1018200 in BORN.AGG_INFANT, or use infant IKN (if available) to link to RPDB to see if DTHDATE in RBPD is within 28 days of B_BDATE in BORN.AGG_INFANT five-minute Apgar score <4 –apgar05_score < 4 in BORN.AGG_INFANT infant resuscitation requiring cardiac compressions—newborn_resuscitation_id = ‘1018620’ in BORN.AGG_INFANT
**Secondary Outcomes**	Severe maternal morbidity or mortality	BIS	Composite binary outcome will be ‘yes’ if one or more of the following: maternal death—Maternal_outcome_id in (‘1016480’, ‘1016470’) from BORN.AGG_PREGNANCY severe pph (defined in row below) pulmonary embolism—lbr_and_birth_complication_id = ‘1014545’ or postpartum_complication_id = ‘1020150’ from BORN.AGG_PREGNANCY amniotic fluid embolism—lbr_and_birth_complication_id = ‘3100949’ or postpartum_complication_id = ‘1020182’ from BORN.AGG_PREGNANCY methicillin-resistant Staphylococcus aureus (MRSA)—postpartum_complication_id = ‘3100564’ from BORN.AGG_PREGNANCY transfer to ICU/CCU—maternal_outcome_id = ‘1016500’ from BORN.AGG_PREGNANCY
	Severe postpartum hemorrhage	BIS	‘Yes’ if one or more of the following PPH and Hysterectomy–(lbr_and_birth_complication_id = ‘1014540’ and lbr_and_birth_complication_id = ‘1014475’) or (lbr_and_birth_complication_id = ‘1014540’ and postpartum_complication_id = ‘1020090’) or (postpartum_complication_id = ‘1020140’ and postpartum_complication_id = ‘1020090’) from BORN.AGG_PREGNANCY PPH and Transfusion—postpartum_complication_id = ‘3100563’ from BORN.AGG_PREGNANCY PPH and Retained placenta surgical removal—(lbr_and_birth_complication_id = ‘1014540’ and lbr_and_birth_complication_id = ‘1014560’) from BORN.AGG_PREGNANCY
	Third- or fourth-degree perineal laceration	BIS	‘Yes’ if perineal_laceration_id in (‘1019960’, ‘1019970’) from BORN.AGG_PREGNANCY
	Emergency services attending the home during labour or the immediate postpartum	BIS	‘Yes’ if any of the following flag variables from BORN.MW_Pregnancy_COC are positive: mw_ems_attend_home_bm mw_ems_attend_home_lbr mw_ems_attend_home_ppm mw_ems_attended_home_lbr_flag
	Stillbirth during the intrapartum	BIS	‘Yes’ if pregnancy_outcome_id = ‘1021090’ from BORN.AGG_INFANT
	Neonatal death	BIS and RPDB	(Defined as death of a liveborn infant in the first 28 completed days of life) ‘Yes’ if either of the following: neonatal_death_id = 1018200 in BORN.AGG_INFANT use infant IKN (if available) to link to RPDB to see if DTHDATE in RBPD is within 28 days of B_BDATE from BORN.AGG_INFANT
	Five-minute Apgar score <4	BIS	‘Yes’ if apgar05_score < 4 from BORN.AGG_INFANT
	Infant resuscitation requiring cardiac compressions	BIS	‘Yes’ if newborn_resuscitation_id = ‘1018620’ from BORN.AGG_INFANT
	Epidural and/or spinal regional analgesia	BIS	‘Yes’ if pain_management_labour_birth_id in (‘1019820’, ‘1019910’, ‘1019920’) from BORN.AGG_PREGNANCY
	Amniotomy	BIS	‘Yes’ if augmentation_id = ‘1012680’ or labour_induction_method_id = ‘1014610’ from BORN.AGG_PREGNANCY
	Oxytocin augmentation of labour	BIS	‘Yes’ if labour_induction_method_id = ‘1014620’ from BORN.AGG_PREGNANCY
	Opiate analgesia	BIS	‘Yes’ if pain_management_labour_birth_id in (‘1019890’) from BORN.AGG_PREGNANCY
	Episiotomy	BIS	‘Yes’ if episiotomy_type_id in (‘1013620’, ‘1013630’) from BORN.AGG_PREGNANCY
	Operative vaginal birth	BIS	‘Yes’ if birth_type_id = ‘1012880’ from BORN.AGG_PREGNANCY
	Caesarean birth	BIS	‘Yes’ if birth_type_id = ‘1012890’ from BORN.AGG_PREGNANCY
	Any breastmilk at 3 days postpartum	BIS	‘Yes’ if mw_newborn_feeding_3_days_id in (‘1027030’, ‘1027040’) from BORN.MW_BIRTH_COC
	Any breastmilk at 10 days postpartum	BIS	‘Yes’ if mw_newborn_feeding_10_days_id in (‘1027030’, ‘1027040’) from BORN.MW_BIRTH_COC
	Any breastmilk at discharge from midwifery care	BIS	‘Yes’ if mw_newborn_feeding_at_discharge_id in (‘1027030’, ‘1027040’) from BORN.MW_BIRTH_COC
	Breastmilk only at 3 days postpartum	BIS	‘Yes’ if mw_newborn_feeding_3_days_id = ‘1027030’ from BORN.MW_BIRTH_COC
	Breastmilk only at 10 days postpartum	BIS	‘Yes’ if mw_newborn_feeding_10_days_id = ‘1027030’ from BORN.MW_BIRTH_COC
	Breastmilk only at discharge from midwifery care	BIS	‘Yes’ if mw_newborn_feeding_at_discharge_id = ‘1027030’ from BORN.MW_BIRTH_COC
**Baseline characteristics**	Maternal Age Category	BIS	If available, use IKN of mother/birthing person to link to RPDB and calculate maternal age on b_bdate from BORN.AGG_PREGNANCY using bdate (of mother/birthing person) from RPDB If not in RPDB, then use maternal birthdate from BORN.AGG_PREGNANCY to calculate age on b_bdate from BORN.AGG_PREGNANCY Categorize as <20, 20–35, and 35+
	Race	BIS	Use Ancestry from BORN.PSOS: • Categorize as Asian, Black, Caucasian, Other, Missing
	Primary Language	BIS	Use primary_language_ID from BORN.AGG_Pregnancy • Categorize as English or French, Other, Missing
	Repeat Ontario midwifery client	BIS	‘Yes’ if the flag variable mw_repeat_client_ON_flag in BORN.MW_Pregnancy_COC is positive; Else ‘No’
	OHIP coverage	RPDB	If available, use IKN of mother/birthing person to link to RPDB and check to see if IKN was valid on b_bdate: if yes, then OHIP coverage = yes; if no, then OHIP coverage = no If no IKN is available, then OHIP coverage = no
	Material Deprivation Quintile (ON MARG)	RPDB; PCCF+; ON MARG	Use the PCCF+ version that is closest to the date of the birth (i.e., the 2011 PCCF+ version for births occurring in 2012–13, the 2016 version for 2014–2018, and the 2021 version for 2019–2021) to assign the DA Obtain postal code from RPDB to link to PCCF+ data & ON MARG Use %getonmarg macro to get quintile value
	Rural residence	RPDB; PCCF+	Link postal code to PCCF+ using the PCCF+ version that is closest to the date of the birth (i.e., the 2011 PCCF+ version for 2012–13, the 2016 version for 2014–2018, and the 2021 version for 2019–2021) • Categorize as rural (population size ≤ 10,000), urban, missing
	Travel time to hospital	RBDB; INST	Distance from patient’s pstlcode on b_bdate from RPDB to the pstlcode of the closest hospital offering 24 hour caesarean section capability; hospitals capacity as defined by the Ontario Provincial Council for Maternal Child Health1; hospital postal code identified in INST; travel time calculated using ArcGIS Categorize as ≤30 minutes or >30 minutes.
	Previous miscarriage/abortion	BIS	Variable num_of_prev_abortions_id in BORN.AGG_Pregnancy No = ‘1019010’ Missing = ‘1019080’ or missing Yes = all other values
	Previous preterm birth	BIS	• Variable num_of_prev_preterm>0 then had_preterm = 1 in BORN.AGG_PREGNANCY
	Grand multiparity	BIS	Variable parity from BORN.AGG_Pregnancy Yes = Parity ≥ 5 No = Parity < 5
	Previous vaginal birth	BIS	Variable num_of_prev_vaginal_births from BORN.AGG_Pregnancy Yes = num_of_prev_vaginal_births > 0 No = num_of_prev_vaginal_births = 0
	Type of conception	BIS	Variable conception_type_ID in BORN.AGG_pregnancy Spontaneous = 1013160 Assisted = 1013130, 1013140, 300006, 1013110, 1013150, 1013120, 1013170 Unknown = 1013180 or missing If both spontaneous and assisted are selected, code as assisted
	Pre-pregnancy Body Mass Index (Kg/m2) group	BIS	Variable maternal_BMI in BORN.AGG_Pregnancy Groups: <18.5; 18.5–24; 25.0–29; 30.0–34; 35.0–39; 40+
	Smoking in pregnancy	BIS	Variable matsmokingatfirstprenvisit in BORN.AGG_Pregnancy No = 1017430 Yes = 1017440, 1017450, 1017460, 1017470 Unknown/missing = 1017475 or missing Variable mat_smoking_at_adm_for_birth in BORN.AGG_Pregnancy No = 1017380 Yes = 1017390, 1017400, 1017410, 1017420 Unknow/missing = 1017425 or missing If mat_smoking_at_adm = yes or matsmokingatfirstprenvisit = yes then mat_smoking_final = 1
	Mental health concerns in pregnancy	BIS	Variable mental_health_concern_ID from BORN.AGG_Pregnancy No = 1017625 Yes = 1017640, 1017660, 1017650, 1017690, 1017680, 1017670, 1017630 Missing = 1017695 or missing
	First trimester prenatal visit	BIS	• Variable first_trimester_visit_flag from BORN.AGG_pregnancy dataset
	Dating ultrasound	BIS	Variable edb_determination_method_ID from BORN.AGG_pregnancy dataset Yes = 1013580, 1013610, 1013600 No = 1013612, 1013590 Missing = 1013615, -1, or missing
	Chronic Anemia	BIS	Variable mat_pre_exist_health_cond_id from BORN.AGG_PREGNANCY Yes = ‘1017030’ or ‘1017060’ No = all other values
	Gestational age ≥ 41+0 weeks	BIS	Variable ga_at_birth_weeks from BORN.AGG_PREGNANCY Yes = ga_at_birth_weeks > 40 No = ga_at_birth_weeks < 41
	Season (Flag for poor weather seasonality)	BIS	Use b-bdate from BORN.AGG_PREGNANCY ‘Yes’ if month of b_bdate in (Nov, Dec, Jan, Feb, Mar) ‘No’ if month of b_bdate in (Apr, May, Jun, Jul, Aug, Sep, Oct)

BIS: Better Outcomes Registry & Network (BORN) Information System; CIHI: Canadian Institute for Health Information; DAD: Discharge Abstract Database; INST: Information about Ontario health care institutions funded by the Ministry of Health or the Ministry of Long-Term Care; OHIP: Ontario Health Insurance Plan; PCCF+: Statistics Canada’s Postal Code^OM^ Conversion File Plus; RPDB: Registered Persons Database.

1. Provincial Council for Maternal and Child Health (PCMCH). Standardized Maternal and Newborn Levels of Care Definitions. Toronto, Ontario, August 1 2013.

### Data sources

We will use several linked provincial-level administrative datasets held by ICES, including prospectively collected perinatal registry data captured by the Better Outcomes and Registry Network (BORN Ontario). Datasets will be linked using unique encoded health identification numbers (the ‘IKN’) and analyzed at ICES. ICES is an independent, not-for-profit organisation whose legal status under Ontario’s Personal Health Information Privacy Act allows it to collect and analyze personal health information without consent for the purpose of health system evaluation and improvement.

The study cohort, as well as variables related to demographic characteristics, obstetric history, antenatal factors, and primary outcomes will be obtained using data from BORN Ontario’s perinatal registry [12]. This is the largest perinatal registry in Canada with nearly complete capture of births in Ontario (approximately 140,000 births/ year; 40% of births in Canada) [12]. Detailed information spanning the antepartum, intrapartum, and postpartum periods is entered into the internet-based BORN Information System (BIS) by care providers at the point of care or uploaded from electronic medical records in hospital, laboratory, and clinic settings across Ontario through. Validation studies of the BIS, including a chart re-abstraction study, have demonstrated good agreement with data from patient charts and with abstracted administrative hospital data [13, 14].

**Fig 2:**
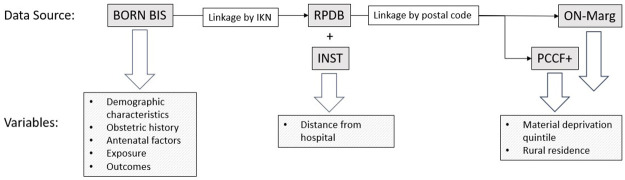
Data linkage schematic.

Other data sources will include the Registered Persons Database (RPDB), which captures demographic information for all residents eligible for the Ontario Health Insurance Plan; the Canadian census and Postal Code Conversion File Plus (PCCF+), which allows census data to be used to create area-level demographic variables through linkage to postal codes; [15] the Ontario Marginalization Index (ON-Marg), a validated index that quantifies the relative level of marginalization of area-level census data; [16] and INST, which is an ICES dataset that includes information about all hospitals in Ontario, including their location. The proposed data linkage schematic is presented in Fig 2.

### Variables

Complete details regarding the data sources and codes for the creation of the exposure, outcomes, and covariates are described in Table 2.

#### Exposure

The exposure will be planned home birth, and the comparator will be planned hospital birth. We will use planned rather than actual place of birth, which is standard in research on home birth outcomes, as it aligns with an intention to treat approach. Emergency, unplanned birth that occurs in the home will be classified under planned hospital birth. We will divide the cohort into three exposure groups—Planned home birth with a midwife, Planned hospital birth with a midwife, and Planned hospital birth with a physician—which are defined below.

Planned home birth with a midwife—This group will include individuals who received a billable course of care from a midwife (at least 12 weeks of midwifery care and/or attendance of a midwife at the birth) and whose BIS records indicate that their planned place of birth at the onset of labour was “home”. Births occurring in a birth center or midwifery clinic (approximately 2% of midwifery births in Ontario) will be excluded from the main analysis. Midwives are the only providers who attend home births in Ontario, and they capture the planned place of birth at the onset of labour in the BORN BIS.

Planned hospital birth with a midwife—This group will include individuals who received a billable course of care from a midwife and the planned place of birth at the onset of labour was hospital.

Planned hospital birth with a physician—This group will include the remaining individuals in the cohort who are not midwifery billable courses of care. These individuals will have received intrapartum care from a family physician or obstetrician.

#### Outcomes

The primary outcome will be severe neonatal morbidity or mortality, a composite binary outcome that includes one or more of the following conditions: stillbirth during the intrapartum period, neonatal death (death of a liveborn infant in the first 28 completed days of life), five-minute Apgar score <4, or infant resuscitation requiring cardiac compressions.

Secondary outcomes will include the individual components of the primary outcome, as well the following additional outcomes:

Adverse maternal outcomes: maternal death; composite severe maternal morbidity (defined as at least one of the following: severe PPH (defined below), pulmonary embolism, amniotic fluid embolism, Methicillin-resistant Staphylococcus aureus (MRSA), or transfer to ICU/CCU); third- or fourth-degree perineal laceration; severe postpartum hemorrhage (defined as PPH and hysterectomy, or PPH and transfusion, or PPH and retained placenta surgical removal); emergency services attending the home during labour or the immediate postpartum.

Obstetric interventions: epidural and/or spinal regional analgesia, amniotomy, oxytocin augmentation of labour, opiate analgesia, episiotomy, operative vaginal birth, caesarean birth.

Newborn feeding (midwifery clients only): any breastmilk at 3 days postpartum, any breastmilk at 10 days postpartum, any breastmilk at discharge from midwifery care, breastmilk only at 3 days postpartum, breastmilk only at 10 days postpartum, breastmilk only at discharge from midwifery care.

#### Covariates

The following demographic characteristics will be obtained from the BIS: age group (>20/20-34/35+), race (Asian/Black/Caucasian/Other/Missing), primary language (English or French/other/missing), and repeat midwifery client (yes/no). We will use data from the RPDB to determine Ontario health insurance plan (OHIP) coverage (yes/no). Individual residential postal codes will be used to derive three other demographic variables: material deprivation quintile (1 (least marginalized), 2, 3, 4, 5 (most marginalized)), rural residency (yes/no), and distance to hospital (≤30 minutes from a hospital with 24/7 caesarean capability/>30 minutes from a hospital with 24/7 caesarean capability). Based on postal code, the Canadian Census and Postal Code Conversion File Plus (PCCF+) will be used to determine participants’ dissemination area (DA), the smallest standard geographic area for which census data are disseminated. We will use the version closest to the date of the birth to assign this (i.e., the 2011 PCCF+ version for 2012–13, the 2016 version for 2014–2018, and the 2021 version for 2019–2021). The Ontario Marginalization Index (ON-Marg), a validated index that quantifies the relative level of marginalization of area-level census data, will be used to determine neighbourhood material deprivation quintile. By linking the study cohort and PCCF+ using residential postal code, and then linking with ON-Marg data, we will obtain ON-Marg quintiles for each individual. We will obtain data on rurality by using PCCF+ to link individual postal codes to census sub-divisions. Rurality will be categorized based on population size ≤ 10,000.

The following characteristics pertaining to obstetric history will be obtained from the BIS: previous miscarriage/abortion (yes/no), previous preterm birth (yes/no), grand multiparity—i.e., ≥5 previous live or stillbirths (yes/no), and previous vaginal birth (yes/no).

We will also use BIS data to include the following characteristics related to the antenatal period: type of conception (assisted/spontaneous), pre-pregnancy BMI group (<18.5/18.5-24/25.0-29/30.0-34/35.0-39/40+), smoking in pregnancy (yes/no), mental health concerns in pregnancy (yes/no), first trimester visit (yes/no), dating ultrasound (yes/no), chronic anemia (yes/no), and gestational age ≥ 41+0 weeks at onset of labour (yes/no).

### Study power

Table 3 presents our estimations of the minimum detectible relative risks for our estimated sample size.

**Table 3:** Estimated minimal detectible relative risks.

*Nulliparous*			
Group	Estimated number of births in the study period (2012–2021)	Minimum detectable RR (vs. planned hospital birth with a physician)	
		Severe neonatal morbidity or mortality (0.4%)	Severe maternal morbidity (1.1%)
Hospital with a physician	187,200	ref	ref
Hospital with a midwife	41,624	1.26	1.16
Home with a midwife	6,776	1.65	1.37
*Parous without a previous caesarean*			
Group	Estimated number of births in the study period (2012–2021)	Minimum detectable RR (vs. planned hospital birth with a physician)	
		Severe neonatal morbidity or mortality (0.4%)	Severe maternal morbidity (1.1%)
Hospital with a physician	255,060	ref	ref
Hospital with a midwife	47,480	1.24	1.14
Home with a midwife	18,465	1.38	1.22
*Parous with a previous caesarean*			
Group	Estimated number of births in the study period (2012–2021)	Minimum detectable RR (vs. planned hospital birth with a physician)	
		Severe neonatal morbidity or mortality (0.7%)	Severe maternal morbidity (1.5%)
Hospital with a physician	25,740	ref	ref
Hospital with a midwife	5,990	1.55	1.37
Home with a midwife	665	3.00	2.25

Our calculations of minimal detectible relative risks are based on the following assumptions:

- We previously identified 80,698 eligible midwife attended births over six years (2012–18). Adding three more years (2018–2021), we estimate there will be at least 121,000 midwife attended births in the cohort.
- Based on proportions observed between 2012–2018, we assume 24% of midwife attended births will be planned to occur at home, and 76% will be planned to occur in hospital. Therefore, we estimate that there will be 29,040 planned home births and 91,960 planned hospital births for midwife attended births.
- Based on the number of physician-attended births between 2012–18, we estimate that there will be about 780,000 physician attended births in 2012–21.
- We estimate that 60% of the physician attended births would be eligible for inclusion in our study, which will result in 468,000 physician attended births.
- We estimated that 40 of the cohort will be nulliparous, 54.5% will be parous with no prior caesarean, and 5.5% will be parous with a previous caesarean
- In the 2016 publication by Hutton et al., the rate of serious neonatal morbidity or mortality was 0.4% [3].
- Reported rates of severe maternal morbidity in Ontario are 1.1% [17].
- We set α = 0.05 and β = 0.8 and assumed a two-tailed test and used SAS to calculate the minimum detectable relative risk.
- We assumed conservatively that only 10% of the variance in the outcome will be explained by covariates. (If greater variance is explained by the covariates, this will reduce the magnitude of the minimum detectable effect (MDE).)

### Data analysis

We will conduct a stratified analysis with strata defined based on a combination of parity and previous caesarean birth, resulting in three strata: nulliparous, parous—no previous caesarean birth, and parous–prior caesarean birth. These strata are important clinically as they are associated with different likelihoods of the outcomes of interest. Presenting results by strata will facilitate meaningful use of the findings in clinical practice, e.g., in discussions with pregnant people about choice of planned place of birth.

We will use descriptive statistics to compare population characteristics according to exposure group within the three strata. Categorical variables will be presented as frequencies and proportions and continuous variables as means or medians (with standard deviation or inter-quartile range.

To reduce the impact of selection bias in estimating the effect of planned place of birth on neonatal and maternal outcomes, we will use propensity score (PS) overlap weighting (OW) as described by Li et al. [18]. This approach uses a propensity score to account for differences between exposure groups in the characteristics associated with the probability of experiencing the exposure of interest (in this case, planning a home birth at the onset of labour). OW assigns weights to each individual in the study that are proportional to the probability of them being in the opposite exposure group. We will use a modified approach for comparisons between three or more groups, which we will operationalize using R Statistical Software with the PSweight package [19].

Separate PS models will be specified for each of the three strata (see Table 4 for lists of the prespecified variables in the PS for each stratum). Selection of the variables for the propensity scores was guided by an extensive literature review of factors associated with planning a home birth and our team’s own multivariate analyses of predictors of planning a home birth among recipients of midwifery care in Ontario. The factors included in the propensity scores are also independently associated with the primary outcome. The balance of covariates between the study groups, stratified by parity and previous caesarean birth, before and after application of PS OW will be assessed by standardized difference, with differences greater than 0.1 considered meaningful [20].

**Table 4:** Variables for propensity scores, by strata.

Variable	Nulliparous	Parous—no prior caesarean	Parous—prior caesarean
Demographic			
Age group	●	●	●
Race	●	●	●
Primary language	●	●	●
Repeat midwifery client	●	●	●
OHIP Coverage	●	●	●
Material deprivation quintile	●	●	●
Rural residency	●	●	●
Distance to hospital	●		●
Obstetric History			
Previous miscarriage/abortion	●	●	●
Previous preterm birth		●	●
Grand multiparity		●	●
Previous vaginal birth			●
Antenatal Factors			
Type of conception	●	●	●
Pre-pregnancy BMI	●	●	●
Smoking in pregnancy	●	●	●
Mental health concerns in pregnancy	●	●	●
First trimester visit	●	●	●
Dating ultrasound	●	●	●
Chronic anemia	●	●	●
Gestational age ≥ 41+0 weeks	●	●	●

Modified Poisson regression with robust standard errors applying the PS OW will then be used to assess the relationship between planned place of birth and study outcomes in separate models, with crude and adjusted risk ratios (RR), corresponding 95% confidence interval (CI) and p-values reported. Repeat pregnancies during the study period will be accounted for in all models including multiparous people by using generalized estimating equations assuming an exchangeable correlation structure [21].

#### Handling of missing data

Records with missing values <3% will be handled using complete case regression analysis. We anticipate that >3% missingness will occur for some covariates, including race, primary language, BMI category, material deprivation quintile, rural, first trimester visit, and gestational diabetes. For these variables we will use the missing indicator approach because missingness reflects important clinical differences in the variables for which we anticipate substantial levels of missing data.

#### Sensitivity analyses

We will undertake two sensitivity analyses to assess the robustness of our results to the methods used for handling missing data. First, we will conduct a complete case analysis by excluding records with missing variables. Second, missing values on covariates with ≥3% missingness will be estimated using multiple imputation by fully conditional specification (10 replications).

In addition to the sensitivity analyses on handling of missing data, one additional sensitivity analysis is planned to assess the robustness of the study findings to residual confounding using E-value methodology, which is the maximal strength of association that an unmeasured confounder would need to have with the exposure and the outcome to fully explain away an observed exposure-outcome association [22].

### Ethics

This project has been reviewed and approved by Hamilton Integrated Research Ethics Board (HiREB)–Project #17343. A privacy review of the proposal has been conducted by ICES. The research protocol has also been reviewed and approved by BORN-Ontario.
